# Identification of Two Lpp20 CD4^+^ T Cell Epitopes in *Helicobacter pylori*-Infected Subjects

**DOI:** 10.3389/fmicb.2018.00884

**Published:** 2018-05-23

**Authors:** Yunshan Ning, Jianbin Ye, Junjie Wen, Danlin Wu, Zhongbiao Chen, Yanqing Lin, Bingxin Hu, Meiqun Luo, Jun Luo, Lijun Ning, Yan Li

**Affiliations:** ^1^School of Laboratory Medicine and Biotechnology, Southern Medical University, Guangzhou, China; ^2^Affiliated Foshan Hospital of Southern Medical University, Foshan, China; ^3^School of Public Health, Southern Medical University, Guangzhou, China

**Keywords:** *Helicobacter pylori*, Lpp20, CD4^+^ T cell, immunodominant epitope, HLA restriction

## Abstract

Antigen-specific CD4^+^ T cells play an essential role in effective immunity against *Helicobacter pylori* (*H. pylori*) infection. Lpp20, a conserved lipoprotein of *H. pylori*, has been investigated as one of major protective antigens for vaccination strategies. Our previous study identified two H-2^d^-restricted CD4^+^ T cell epitopes within Lpp20 and an epitope vaccine based on these epitopes was constructed, which protected mice in prophylactic and therapeutic vaccination against *H. pylori* infection. Immunodominant CD4^+^ T cell response is an important feature of antiviral, antibacterial, and antitumor cellular immunity. However, while many immunodominant HLA-restricted CD4^+^ T cell epitopes of *H. pylori* protective antigens have been identified, immunodominant HLA-restricted Lpp20 CD4^+^ T cell epitope has not been elucidated. In this study, a systematic method was used to comprehensively evaluate the immunodominant Lpp20-specific CD4^+^ T cell response in *H. pylori*-infected patients. Using *in vitro* recombinant Lpp20 (rLpp20)-specific expanded T cell lines from *H. pylori*-infected subjects and 27 18mer overlapping synthetic peptides spanned the whole Lpp20 protein, we have shown that L_55–72_ and L_79–96_ harbored dominant epitopes for CD4^+^ T cell responses. Then the core sequence within these two 18mer dominant epitopes was screened by various extended or truncated 13mer peptides. The immunodominant epitope was mapped to L_57–69_ and L_83–95_. Various Epstein-Barr virus (EBV) transformed B lymphoblastoid cell lines (B-LCLs) with different HLA alleles were used as antigen presenting cell (APC) to present peptides to CD4^+^ T cells. The restriction molecules were determined by HLA class-antibody blocking. L_57–69_ was restricted by DRB1-1501 and L_83–95_ by DRB1-1602. The epitopes were recognized on autologous dendritic cells (DCs) loaded with rLpp20 but also those pulsed with whole cell lysates of *H. pylori* (HP-WCL), suggesting that these epitopes are naturally processed and presented by APC. CD4^+^ T cells were isolated from *H. pylori*-infected patients and stimulated with L_57–69_ and L_83–95_. These two epitopes were able to stimulate CD4^+^ T cell proliferation. This study may be of value for the future development of potential *H. pylori* vaccine.

## Introduction

*Helicobacter pylori* (*H. pylori*) infects more than half of the population in the world. The infection is causally associated with gastritis, peptic ulcer, gastric adenocarcinoma and gastric mucosa-associated lymphoid tissue lymphoma. A growing body of evidence shows that CD4^+^ T cell response plays a vital role in protective immunity against *H. pylori*. An increased number of T cells were infiltrating in human stomach with a typical Th1 phenotype during *H. pylori* infection (Bamford et al., 1998). A predominant Th1-type response was also elicited early during *H. pylori* infection in rhesus macaques (Mattapallil et al., 2000). Either natural infection or vaccine-induced immunity to *H. pylori* depends on a strong Th1-type cellular adaptive immune response in mice (Ermak et al., 1998; Eaton et al., 2001; Akhiani et al., 2002). These studies demonstrate that the protective adaptive immunity against *H. pylori* involves in Th1 response.

Immunodominance is the phenomenon in which the cellular immune response tends to focus on only a few of antigenic epitopes even during responses to complex antigens or pathogens in infected or immunized individuals. Generally, immunodominant T cells are more prevalent and protective in immune response compared with subdominant ones. Therefore, the immunodominant T cells often provide effective immune response and play a pivotal role in the adaptive immunity against pathogens, and this has been well-demonstrated in many bacterial, viral, and tumor systems (Jackson et al., 2006; Wu et al., 2011). Many scientists in the vaccine field think that immunodominant CD4^+^ T cell epitopes seem to be critical for *H. pylori* vaccine development (Ermak et al., 1998; Akhiani et al., 2002; Nyström and Svennerholm, 2007). Although several immunodominant epitopes of *H. pylori* protective antigens were identified (Chen et al., 2013; Yang et al., 2013; Hu et al., 2016), few are known to immunodominant epitope-specific CD4^+^ T cells response of other *H. pylori* antigen and immunodominant CD4^+^ T cell epitope has yet to be elucidated.

Lpp20, an outer membrane lipoprotein on *H. pylori*, has been considered to be one of potential vaccine candidates (Kostrzynska et al., 1994; Keenan et al., 2000; Peter and Beglinger, 2007; Li et al., 2012). Our previous study identified one Lpp20 B cell epiotope (L_108–119_) which induced mouse anti-Lpp20 serum (Li et al., 2007) and two H-2^d^-restricted Lpp20 CD4^+^ T cell epitopes (L_58–72_ and L_83–97_) which elicited Th1-type immune response in BALB/c mice (Li et al., 2012). Additionally, the epitope vaccine composed of above mentioned three epitopes could stimulate the production of high level of Lpp20-specific antibodies and Th1-type cytokines and significantly reduced *H. pylori* colonization in *H. pylori*-challenged mice (Li et al., 2015), suggesting that Lpp20-derived epitope-based vaccine could be a promising alternative to eradicate *H. pylori* infection. Due to the MHC molecule differences in mice and humans, H-2^d^-restricted CD4^+^ T cell epitopes cannot sometimes induce effective immune responses in humans. Thus, the identification of such epitopes might be important for the future development of potential *H. pylori* vaccine.

In the present study, we conducted a systematic mapping analysis to screen *H. pylori* Lpp20 immunodominant CD4^+^ T cell epitopes using *in vitro* expanded recombinant Lpp20 (rLpp20)-specific T cell lines from *H. pylori*-infected subjects and 27 18mer overlapping synthetic peptides covered the sequence of Lpp20 protein. We observed that CD4^+^ T cell responses to Lpp20 varied remarkably with broad epitope specificity and the main responses focused on L_55–72_ and L_79–96._ Further, the core sequence of 18mer immunodominant epitopes was identified by 13mer overlapping peptides and various truncated or extended peptides based on the 13mer peptide. The immunodominant epitope was mapped into L_57–69_ and L_83–95_. The former epitope was restricted by HLA-DRB1^*^1501 and the latter one was restricted by HLA-DRB1^*^1602 and they were both naturally processed and presented by APC.

## Materials and methods

### Subjects and blood samples

This study was approved by the Institutional Human Ethics Review Board of Nanfang Hospital, Southern Medical University, Guangzhou, China and carried out in accordance with the recommendations. All subjects gave written informed consent in accordance with the Declaration of Helsinki. Gastric diseases were diagnosed by both endoscopic and histopathologic examination. ^13^C Urea breath test and serum anti-*H. pylori* antibody ELISA were performed to screen *H. pylori*-infected subjects. Blood samples from these subjects who donated more than 200 mL blood were collected and peripheral blood mononuclear cells (PBMCs) were isolated by Ficoll-Paque™ (GE Healthcare) gradient and then stored in liquid nitrogen until use. The HLA genotype of PBMC was determined by polymerase chain reaction (PCR) with sequencing-based typing at Beijing Genomics Institute (BGI), Shenzhen, China.

### rLpp20 antigen and Lpp20 synthetic peptides

**Figure 1:**
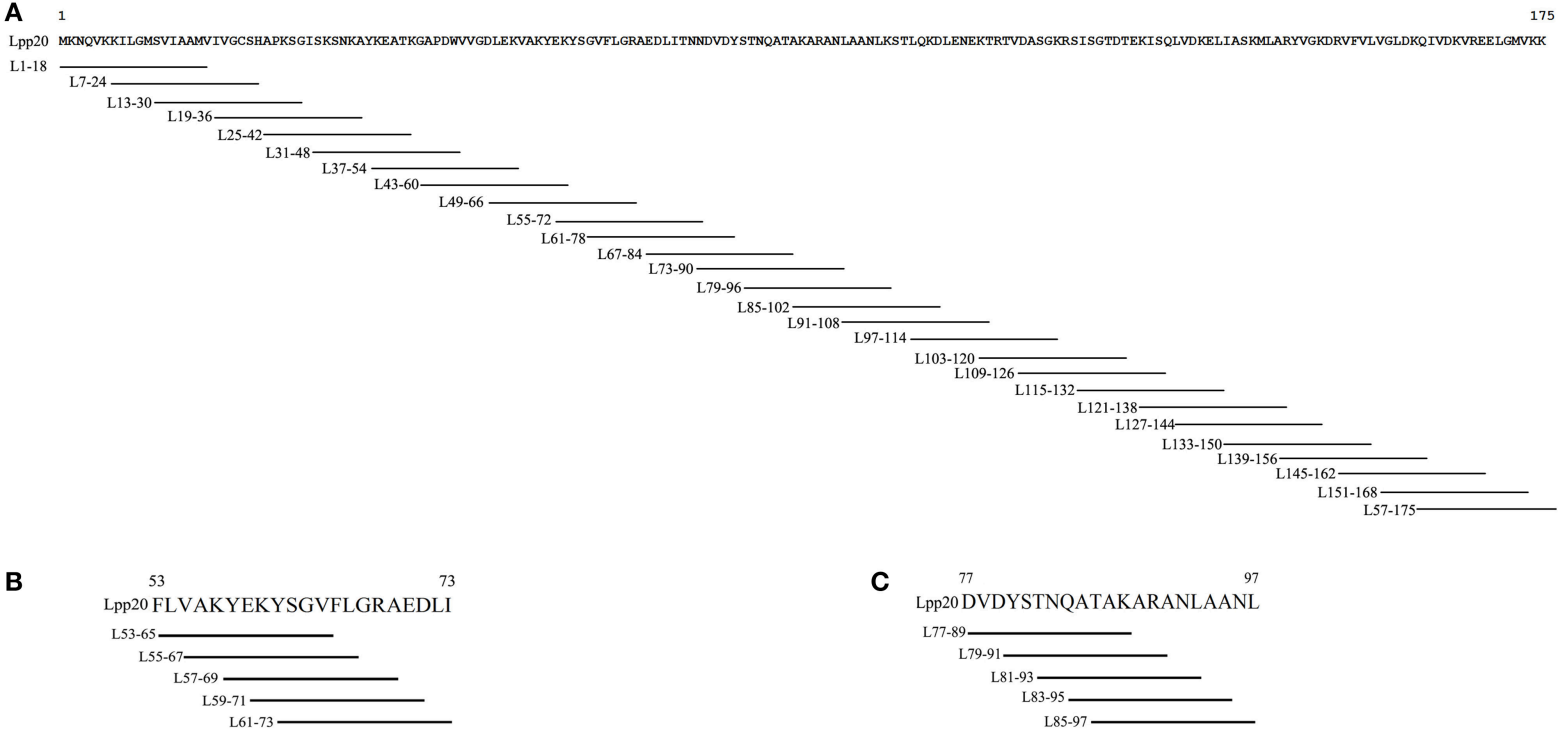
The schematic diagram of 18mer and 13mer overlapping peptides covering Lpp20 protein **(A)** 18mer synthetic peptides that covered the whole Lpp20 protein and overlapped by 12 amino acids. **(B)** Various N- and C-terminus extended or truncated 13mer peptides were based on initially identified dominant 18mer L_55–72_ and overlapped by 11 amino acids. **(C)** Various N- and C-terminus extended or truncated 13mer peptides were based on initially identified dominant 18mer L_79–96_ and overlapped by 11 amino acids.

rLpp20 was expressed in *Escherichia coli* and purified as we described previously (Li et al., 2007) and stored at −70°C. Amino acid sequence of Lpp20 has been submitted to NCBI by us (No. AAZ13599). 18mer synthetic peptides that covered the whole Lpp20 protein and overlapped by 12 amino acids (Figure 1A) and 13mer peptides that covered the initially identified dominant 18mer sequence and overlapped by 11 amino acids (Figures 1B,C) were synthesized and purified (purity >95%) by GL Biochem (Shanghai, China). All synthetic peptides were dissolved in dimethyl sulfoxide (DMSO, Sigma, Shanghai, China) and stored at −80°C.

### The expansion of Lpp20-specific CD4^+^ T cells from *H. pylori*-infected subjects *in vitro*

PBMCs (1–2 × 10^6^) were pulsed with 0.2 μmol/L Lpp20 (or 5 μmol/L immunodominant peptide) and cultured in RPMI 1640 medium (Gibco) supplemented with 5% human AB sera, 2-mercaptoethanol (5 × 10^−5^ mol/L), L-glutamine (2 mmol/L), and antibiotics (penicillin 100 U/mL and streptomycin 100 μg/mL) in 48-well cell culture plates. The culture medium was 50% replaced by the above-mentioned medium containing 10 U/mL recombinant human interleukin-2 (rh IL-2) on day 5 and then 50% replaced by above medium containing 25 U/mL rhIL-2 when the color of the medium was yellow. The cells were split on day 10 and harvested on day13.

### Systematic identification of immunodominant Lpp20 CD4^+^ T cell epitopes

PBMCs were thawed and stimulated with rLpp20. After 13 days, T cells were screened against 27 18mer overlapping peptides individually. The intracellular cytokine staining (ICS) and flow cytometry were used to analyze the frequency of IFN-γ-secreting CD4^+^ T cells. Further, the same method was used to determine the core sequence of 18mer immunodominant CD4^+^ T cell epitopes using 13mer overlapping peptides.

### Establishment of epstein-barr virus (EBV) transformed B lymphoblastoid cell lines (B-LCLs)

Using the culture supernatant from EBV-producing B95-8 cells, B lymphoblastoid cell lines (B-LCLs) were established from autologous PBMCs and cultured in RPMI-1640 (GIBCO) supplemented with 10% fetal calf serum (GIBCO), 2-ME (5 × 10^−5^ M), L-glutamine (2 mM), and antibiotics (penicillin 100 U/ml and streptomycin 100 μg/ml).

### Intracellular cytokine staining (ICS)

In the assays for screening immunodominant CD4^+^ T cell epitopes by 18mer and 13mer synthetic peptide, bulk cultured Lpp20-specific T cells were incubated with 5 μmol/L peptide at 37°C for 5 h in the presence of monensin (Becton Dickinson, Shanghai, China). In the assays for determining HLA-restricting alleles, B-LCLs were incubated with 5 μmol/L peptide for 1 h and then the free peptides were washed out. After that, the B-LCLs were co-cultured with Lpp20-specific T cells at a ratio of 1:10 (B-LCLs/T cells) for 5 h in the presence of monensin. In the antibody-blocking assay, APCs were incubated with 5 μg/mL antibodies against HLA-DP (Abcam), HLA-DR (Biolegend), and HLA-DQ (Biolegend) for 30 min before addition of peptide and monensin. After then, T cell activation was assessed by ICS. The cells were harvested and stained firstly with anti-CD3-PE and anti-CD4-APC (Biolegend, Beijing, China) in 50 μl staining buffer (PBS containing 1% heat inactivated FCS) at 4°C for 30 min, washed, fixed with Fixation/Permeabilization solution (BD Cat. No.554715) at 4°C for 20 min and stained with anti-IFN-γ-FITC (Biolegend, Beijing, China) according to the instruction of the kit protocol. 1 × 10^5^ cells were acquired on a FACSCanto II flow cytometer (Becton Dickinson). Lymphocytes were gated by forward scatter (FSC) and side scatter (SSC). CD4^+^ T cells subsets were further identified by CD3-PE and CD4-APC staining. Finally, gated CD4^+^ T cells were analyzed for IFN-γ-FITC. FACS data were analyzed using FlowJo software (Tree Star, Inc. Ashland, OR, USA).

### Preparation of *H. pylori* whole cell lysates (HP-WCL)

As previously described (Taylor et al., 2006), *H. pylori* NCTC11637 strain was grown on brain-heart infusion (BHI) plates containing 7% goat blood, trimethoprim (5 μg/mL), polymyxin B (5 μg/mL), and vancomycin (10 μg/mL) and propagated in Brucella broth with 5% fetal bovine serum with gentle shaking at 37°C under microaerobic conditions (85% N_2_, 10% CO_2_, 5% O_2_,). After being cultured for 1 day, bacteria were collected, washed, and lysed. The lysates were centrifuged at 10,000 *g* for 20 min at 4°C to remove intact cells and large debris. The supernatant was sonicated as *H. pylori* whole cell lysates (HP-WCL) as previously described (Chen et al., 2004). The protein content was measured using the BCA Protein Assay Kit (Beyotime, Shanghai, China). All lysates were aliquoted and stored at −20°C until future use.

### Generation of dendritic cells (DCs) and co-culture with immunodominant epitope-specific T cells

PBMCs were thawed and CD14^+^ cells were isolated from PBMCs using CD14 immunomagnetic beads (Miltenyi Biotec, Shanghai, China). Then the cells were cultured in RPMI 1640 medium (Gibco) supplemented with 5% AB human sera, 10 ng/mL interleukin-4, and 20 ng/mL granulocyte-macrophage colony-stimulating factor (GM-CSF) at 37°C in a 5% CO_2_ incubator. On day 6, dendritic cells (DCs) were harvested and pulsed with immundominant peptides, rLpp20, HP-WCL, and bovine serum albumin (BSA) as a negative control at a final concentration of 50 μg/mL for 24 h. Then, DCs and epitope-specific T cells were co-cultured at a ratio of 1:10 for 5 h in the presence of monensin.

### CD4^+^ T cell proliferation assay

CD4^+^ T cells (2 × 10^5^) isolated from *H. pylori*-infected patients were incubated in medium alone or in the presence of phytohemagglutinin (PHA, 5 μg/ml) to assess cell vitality, or in the presence of peptides (20 μg/ml). Cultures were incubated in a total volume of 200 μl for 3 days (37°C, 5% CO_2_) and pulsed during the last 18 h with tritiated thymidine [^3^H] (1 μCi/well). [^3^H] thymidine incorporation was measured in a liquid scintillation counter after collecting cells onto glass fiber filters. The stimulation index (SI) was determined by comparing ^3^H thymidine incorporation in the peptide-stimulated wells with unstimulated wells using the following equation: SI = mean cpm of peptide wells/mean cpm of no peptide wells. Experiments were independently repeated three times.

### Statistical analysis

The Student's *t*-test was used to analyze the differences between two groups. Welch's correction was applied when the variances of two compared groups were not equal. Differences were considered to be significant when the *P*-value was < 0.05.

## Results

### The frequency of Lpp20-specific CD4^+^ T cells in *H. pylori*-infected subjects was higher than that in uninfected-subjects

**Figure 2:**
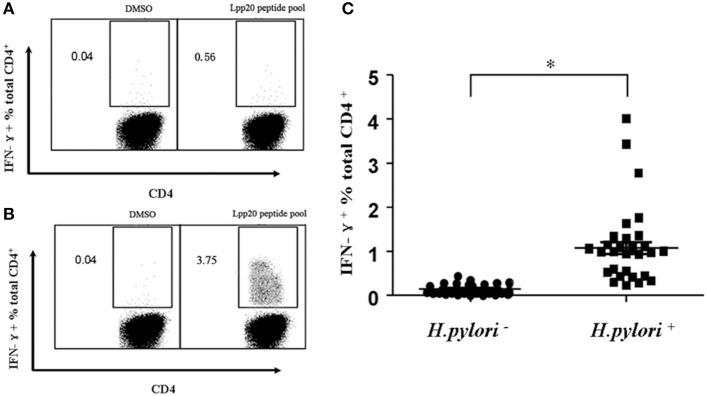
Lpp20-specific CD4^+^ T cell responses in *H. pylori*-infected individuals. PBMCs from **(A)** *H. pylori*-uninfected subject N1 or **(B)** *H. pylori*-infected subject H1 were stimulated with recombinant Lpp20 and the percentage of IFN-γ producing antigen-specific CD4^+^ T cells were determined in an ICS assay using the Lpp20 peptide pool on day 13. DMSO was used as a control. **(C)** PBMCs from 30 *H. pylori* infection-negative (–) and 30 *H. pylori* infection-positive (+) subjects were stimulated with recombinant Lpp20 and the percentage of IFN-γ producing CD4^+^ T cells were assessed as previously mentioned. ^*^p < 0.001.

To assess Lpp20-specific CD4^+^ T cell responses in *H. pylori-*infected subjects, PBMCs were isolated from *H. pylori*-infected subjects and the frequency of Lpp20-specific CD4^+^ T cells was determined by ICS after incubation with pooled 18mer peptides spanning the entire Lpp20 protein. However, the frequency of Lpp20-specific CD4^+^ T cells was too low to be detected *ex vivo* (Figure 2A). Specific cell expansion is typically required to low frequency responses, especially in the epitope mapping (Sayi et al., 2009). Therefore, Lpp20-specific CD4^+^ T cells were expanded by pulsing PBMCs with rLpp20. As a result, the frequency of the Lpp20-specific CD4^+^ T cells became higher after *in vitro* stimulation (Figure 2B). To further confirm the presence of Lpp20-specific CD4^+^ T cells in *H. pylori*-infected subjects, PBMCs from 30 *H. pylori*-infected and 30 uninfected subjects were expanded and then the frequency of Lpp20-specific CD4^+^ T cells were measured as mentioned above. It was shown that the frequency of Lpp20-specific CD4^+^ T cells in *H. pylori*-infected subjects was significantly higher than that in uninfected-subjects (Figure 2C). These results indicate that the stimulation with rLpp20 was able to reactivate antigen-experienced CD4^+^ T cells *in vivo*.

### L_55–72_ and L_79–96_ were two dominant regions recognized by Lpp20-specific CD4^+^ T cells

**Figure 3:**
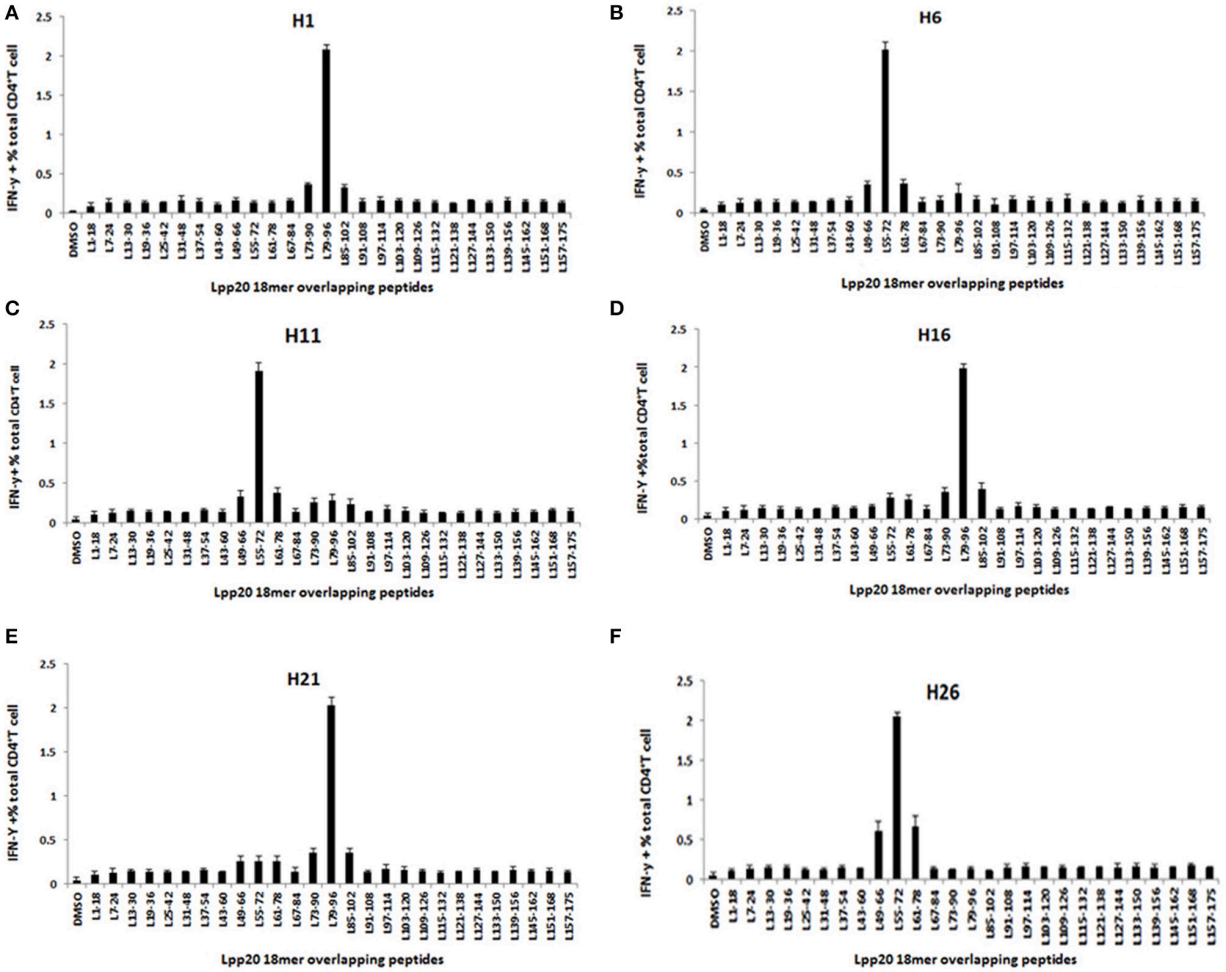
The mapping and identification of the immunodominant Lpp20 CD4^+^ T cell epitope. Lpp20-specific T cells from *H. pylori*-infected subject were expanded *in vitro* as described above and further screened for their specific response to the 27 Lpp20 18mer overlapping peptides at a final concentration of 5 μmol/L in an ICS assay. The identified 18mer sequences are shown **(A–F)** correspond to subjects H1, H6, H11, H16, H21, and H26, respectively.

To systematically investigate the fine specificity and full breadth of Lpp20-specific CD4^+^ T cell responses, PBMCs from several *H. pylori*-infected subjects were stimulated with rLpp20 and T cells were screened against 27 18mer overlapping peptides individually after 13 days. As shown in Figure 3, the Lpp20-specific CD4^+^ T cells recognized two dominant regions (L_55–72_ and L_79–96_). Lpp20-specific CD4^+^ T cells from subjects H6, H11, and H26 mainly focused on L_55–72_ (Figures 3B,C,F), whereas T cells from subjects H1, H16, and H21 recognized L_79–96_ (Figures 3A,D,E). Taken together, L_55–72_ and L_79–96_ contain immunodominant CD4^+^ T cell epitopes of Lpp20.

### L_57–69_ and L_83–95_ were the core sequences of immunodominant epitope within L_55–72_ and L_79–96_, respectively

**Figure 4:**
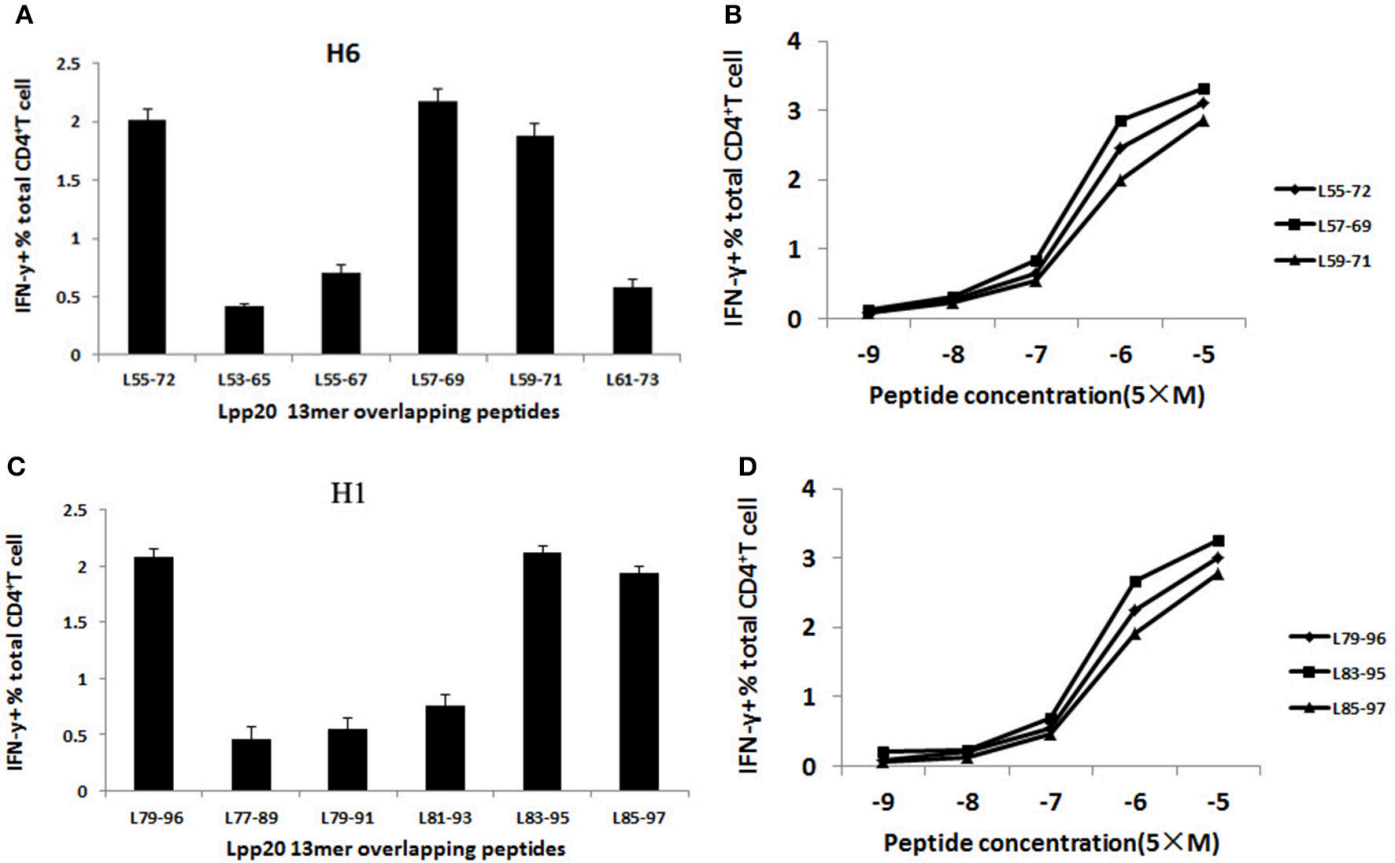
The identification and characterization of the core sequence of L_55–72_ and L_79–96_ immunodominant epitope. Lpp20-specific T cells from subject H1 and H6 were expanded *in vitro* as described above. The cells were screened for their specific response to the 5 Lpp20 13mer overlapping peptides (final concentration ~5 μmol/L) in an ICS assay. **(A)** The 13mer overlapping peptides within the L_55–72_ 18mer epitope were screened with L_55–72_ specific T cells. **(B)** The 13mer overlapping peptides within the L_79–96_ 18mer epitope were screened with L_79–96_ specific T cells. **(C)** Three of the reactive peptide L_55–72_, L_57–69_, and L_59–71_ were titrated to compare their activity. **(D)** Three of the reactive peptide L_79–96_, L_83–95_, and L_85–97_ were titrated to compare their activity.

To further and systemically characterize the core sequence within the immunodominant Lpp20 CD4^+^ T cell epitope-containing peptides L_55–72_ and L_79–96_, T cells reactive to these two peptides were expanded as mentioned above and assessed using a set of 13mer overlapping peptides covering the initially detected 18mer peptides and various N- and C-terminus extended or truncated peptides based on the dominant 18mer sequence (Figures 1B,C). For subject H6, the L_55–72_-specific CD4^+^ T cells mainly recognized L_57–69_ and L_59–71_ (Figure 4A). L_57–69_ stimulated more T cells equivalent to the L_55–72_ response, suggesting that this 13mer peptide was the most potent core sequence of the immunodominant Lpp20 CD4^+^ T cell eptiope in subject H6 (Figure 3A). In addition, this was further confirmed by titration of L_55–72_, L_57–69_, and L_59–71_ at a concentration of 5 × 10^−9^ mol/L−5 × 10^−5^ mol/L (Figure 4B). For subject H1, the L_79–96_-specific CD4^+^ T cells mainly focused on L_83–95_ and L_85–97_ (Figure 4C). L_83–95_ stimulated more T cells equivalent to the L_79–96_ response, suggesting that this 13mer peptide was the most potent core sequence of the immunodominant Lpp20 CD4^+^ T cell eptiope in subject H1 (Figure 4C). Moreover, this was further confirmed by titration of L_79–96_, L_83–95_ and L_85–97_ at a concentration of 5 × 10^−9^ mol/L−5 × 10^−5^ mol/L (Figure 4D). Taken together, the most potent immunodominant CD4^+^ T cell epitopes of Lpp20 were L_57–69_ and L_83–95_.

### L_57–69_ and L_83–95_-responding CD4^+^ T cells were restricted by HLA-DRB1^*^1501 and HLA-DRB1^*^1602, respectively

**Figure 5:**
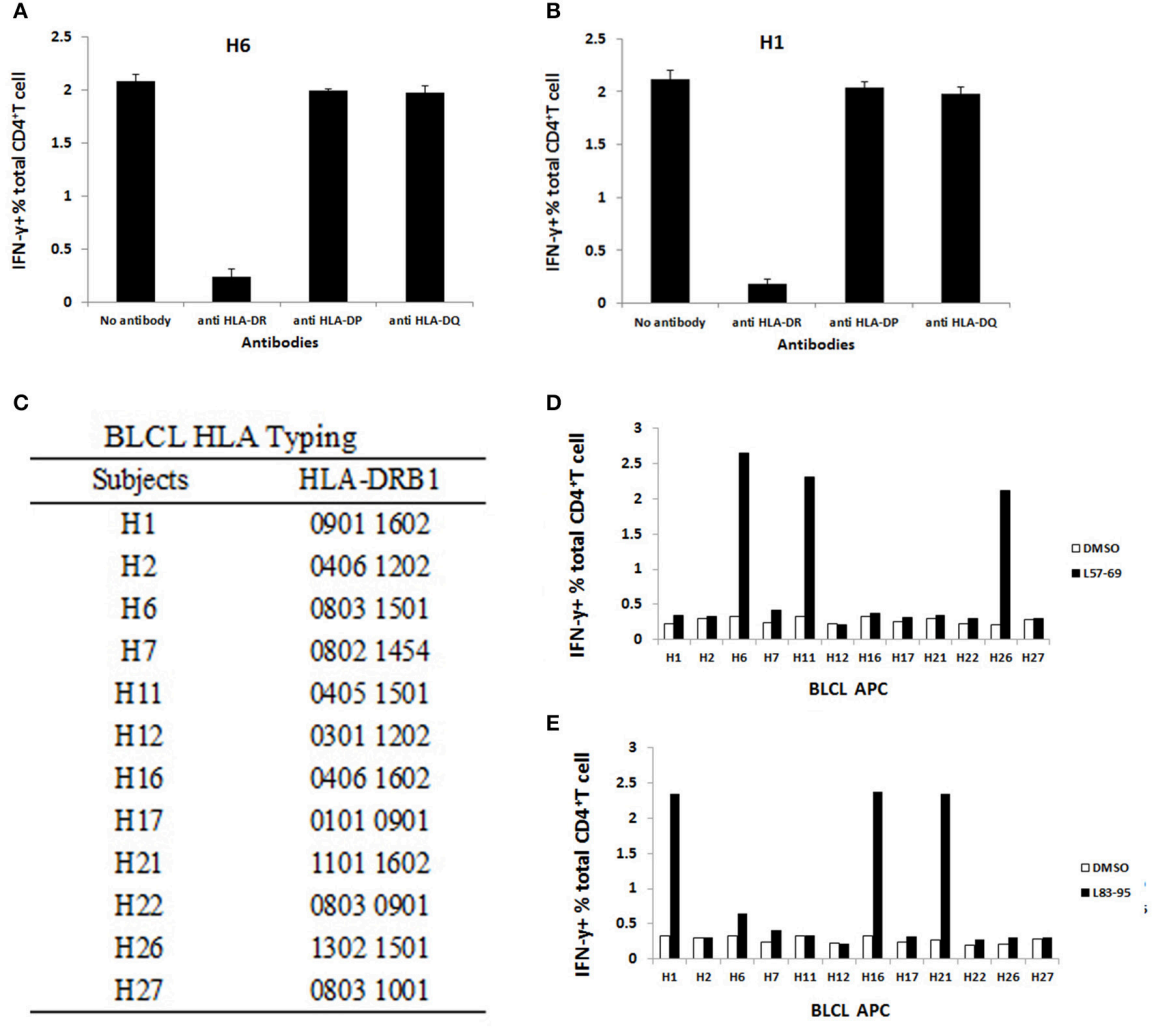
Peptides alone or in presence of blocking antibodies against HLA-DR, HLA-DP, or HLA-DQ. HLA-II antibodies were used to identify the HLA locus presenting the L_57–69_ **(A)** and L_83–95_ **(B)** 13mer peptide. The results were shown as average frequency ± standard deviation (*n* = 3). Only anti-HLA DR antibodies abrogated the responses, demonstrating that the responses to L_55–69_ and L_83–95_ are MHC Class II restricted. **(C)** A panel of B-LCLs with different DR types was identified. HLA-matched B-LCLs were used to further identify the HLA molecule presenting the 13mer peptide L_57–69_ **(E)** and L_83–95_ **(D)**.

To determine the restricting HLA molecule of L_57–69_ and L_83–95_, a MHC Class-II antibody-blocking assay was operated. PBMCs obtained from subject H6 was pulsed with L_57–69_ and cultured in the presence of HLA-DR, HLA-DP, or HLA-DQ blocking antibodies and ICS assays were performed. As shown in Figure 5A, the anti-HLA-DR antibody inhibited IFN-γ-secretion in response to L_57–69_, whereas the anti-DP and anti-DQ antibodies did not. The same method was also used to determine the HLA restriction of L_83–95_. PBMCs obtained from subject H1 was pulsed with L_83–95_ and cultured in the presence of HLA-DR, HLA-DP, or HLA-DQ blocking antibodies and then determined in ICS assays. The HLA-DR antibody blocking inhibited IFN-γ-secretion in response to L_83–95_, whereas the HLA-DP and HLA-DQ blocking did not (Figure 5B). Thus, the L_57–69_ and L_83–95_ epitopes appear to be restricted by HLA-DR molecules.

To further define the HLA-DR allele restricting L_57–69_, a panel of B-LCLs with different HLA-DR genotypes (Figure 5C) was pulsed with L_57–69_ as APCs to stimulate the peptide-specific T cells. As shown in Figure 5D, autologous B-LCL from subject H6 efficiently activated L_57–69_-specific T cells whereas the B-LCL from subject H22 and H27 expressed the same HLA-DRB1^*^0803 but did not present this peptide. Therefore, L_57–69_ must be restricted by HLA-DRB1^*^1501. Similarly, the B-LCL from subject H11 and H26 expressed HLA-DRB1^*^1501 could stimulate L_57–69_-specific T cells. The same method was used to determine the HLA-DR restriction of L_83–95_. A panel of B-LCLs with different HLA-DR genotypes was pulsed with L_83–95_ as APCs to stimulate the peptide-specific T cell line. As shown in Figure 5E, autologous B-LCL from subject H1 efficiently induced L_57–69_-specific T cells. In contrast, the B-LCL from subject H17 and H22 expressed the same HLA-DRB1^*^0901 but did not present this peptide. Therefore, L_57–69_ must be restricted by HLA-DRB1^*^1602.

### L_57–69_ and L_83–95_ were naturally processed and presented by APCs

**Figure 6:**
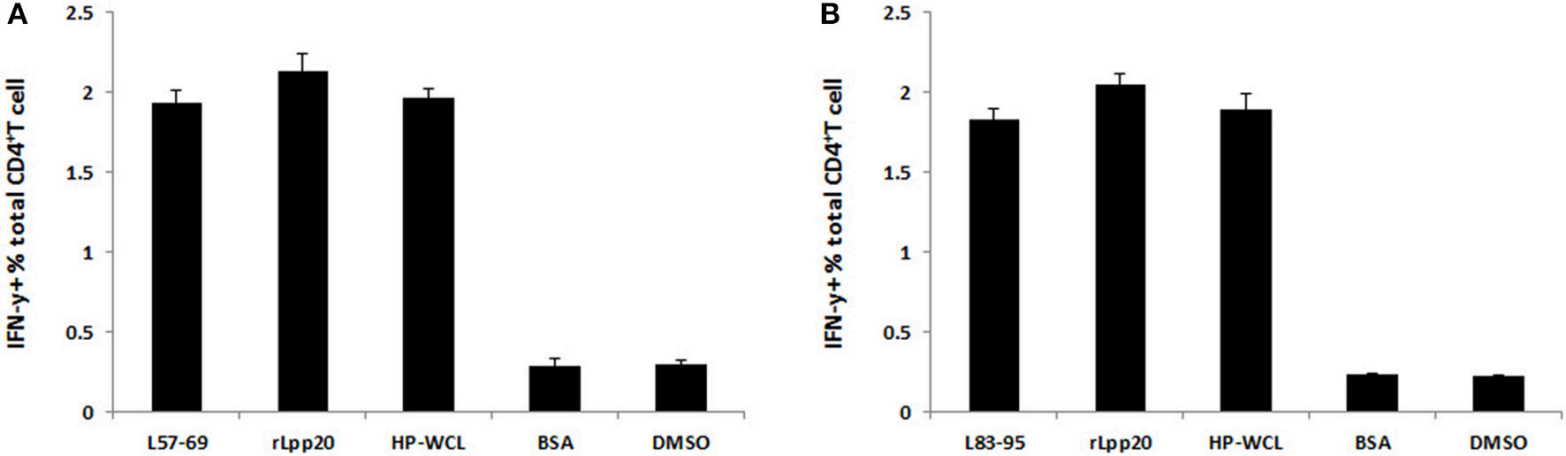
The natural processing and presentation of L_57–69_ and L_83–95_ by DCs. **(A)** The DCs were pulsed with L_57–69_, rLpp20, HP-WCL, or BSA for 24 h and then co-cultured with L_57–69_-specific T cells from subject H6 for 5 h in the presence of monensin. The frequency of IFN-γ-secreting CD4^+^ T cells was determined by ICS. **(B)** L_83–95_-specific T cells from subject H1 were used to determine in a manner similar to the peptide described in **(A)**.

To evaluate whether L_57–69_ and L_83–95_ were naturally presented by APCs, autologous DCs were loaded with rLpp20, HP-WCL, BSA or an immunodominant epitope peptide for 24 h and then co-cultured with epitope-specific T cells for 5 h in the presence of monensin. ICS was carried out to evaluate whether the corresponding epitope-specific T cells could recognize these APCs. As shown in Figure 6A, pulsing the DCs of subject H6 with L_57–69_, Lpp20, HP-WCL vigorously stimulated the L_57–69_-expanded T cell line. However, the DCs pulsed with BSA or DMSO only elicited the L_55–72_-specific CD4^+^ T cells to background levels. Similarly, we also confirmed that L_79–96_ could be naturally processed and presented by autologous DCs (Figure 6B).

### L_57–69_ and L_83–95_ stimulated CD4^+^ T cell proliferation

**Figure 7:**
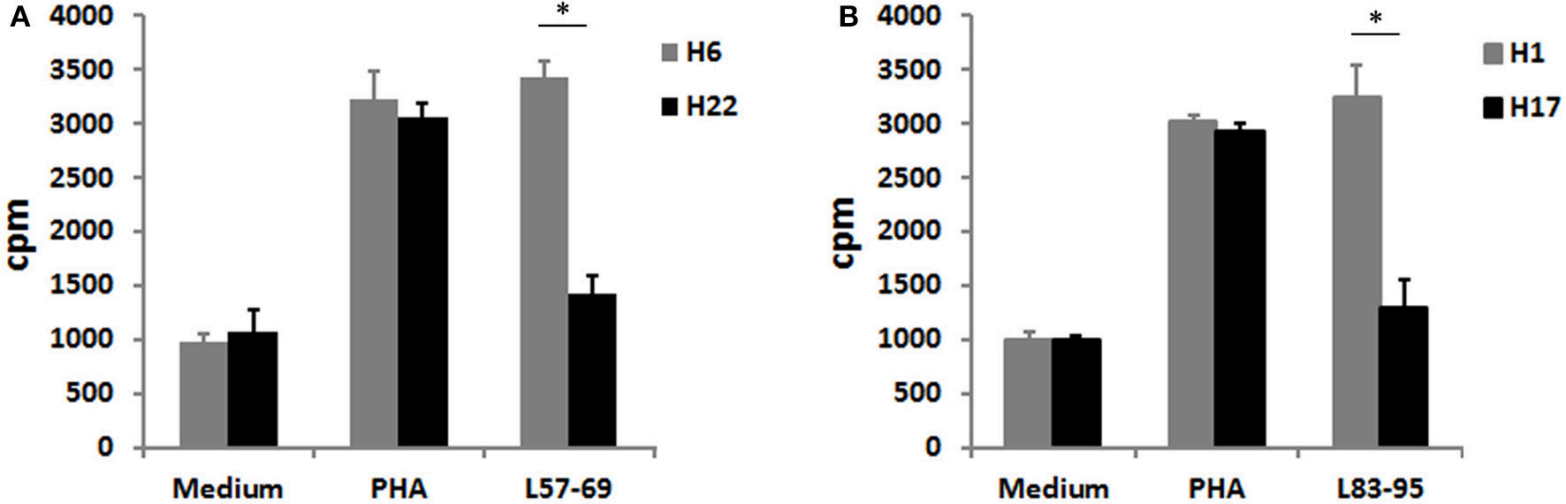
**(A)** CD4^+^ T cells isolated from *H. pylori*-infected subject H6 and H22 were tested in proliferative responses to peptide L_57–69_ (20 μg/ml), phytohemagglutinin (PHA, 5 μg/ml), and control medium. **(B)** CD4^+^ T cells isolated from *H. pylori*-infected subject H1 and H17 were tested in proliferative responses to peptide L_83–95_ (20 μg/ml), rLpp20 (5 μg/ml), and control medium. CD4^+^ T cells treated with PBS were served as negative controls. CD4^+^ T cells treated with PHA were served as positive controls. All data were reported as means ± *SD* of three experiments. ^*^p < 0.05 vs. control.

To evaluate whether L_57–69_ and L_83–95_ stimulate CD4^+^ T cell proliferation, CD4^+^ T cells isolated from *H. pylori*-infected subjects were stimulated with L_57–69_ and L_83–95_. As shown in Figure 7A, L_57–69_ stimulated the proliferation of CD4^+^ T cell isolated from *H. pylori*-infected subject H6 expressed HLA-DRB1^*^ 0803 1501, but couldn't stimulate CD4^+^ T cell isolated from *H. pylori*-infected subject H22 expressed HLA-DRB1^*^0803 0901. As shown in Figure 7B, L_83–95_ stimulated the proliferation of CD4^+^ T cell isolated from *H. pylori*-infected subject H1 expressed HLA-DRB1^*^0901 1602, but couldn't stimulate CD4^+^ T cell isolated from *H. pylori*-infected subject H17 expressed HLA-DRB1^*^0101 0901. Taken together, L_57–69_ and L_83–95_ could stimulate the proliferation of CD4^+^ T cells from *H. pylori*-infected subjects expressed HLA-DRB1^*^1501 and HLA-DRB1^*^1602, respectively.

## Discussion

Increasing evidence shows that antigen-specific CD4^+^ T cell response plays an essential role in anti-*H. pylori* protective immunity (D'Elios et al., 1997a; Sayi et al., 2009; Sjokvist Ottsjo et al., 2015). More importantly, the immunodominant CD4^+^ T cell response is considered to be more effective than subdominant response in the host adaptive immune response to *H. pylori* infection (Ermak et al., 1998; Akhiani et al., 2002; Nyström and Svennerholm, 2007). The type of CD4^+^ T cell response against *H. pylori* may vary according to the antigen involved (D'Elios et al., 1997b). Although some previous studies on *H. pylori*-specific Th1 cell responses focused on specific antigens, they did not identify individual epitopes and therefore were not aware of the influence of involved HLA (D'Elios et al., 1997b, 2003). Recently, many scientists began to pay attention to identifying HLA-restricted CD4^+^ T cell epitopes of *H. pylori* protective antigens using overlapping synthetic peptides, such as UreB_373−385_ and UreB_438−452_ (Yang et al., 2013), HpaA_88−100_ and HpaA_142−159_ (Chen et al., 2013; Hu et al., 2016). Chen et al. found that the HpaA_88−100_-specific Th1-polarized response in *H. pylori*-infected subjects was linked with resistance to severe *H. pylori-*associated gastric diseases (Chen et al., 2013). This kind of protection was also supported by epidemiologic and genetic studies. These findings suggest that efficient presentation and recognition of some *H. pylori* antigens by T cell receptor (TCR) could influence the outcome of associated pathologies. Thus, further studies on antigen processing, presentation, and TCR recognition during *H. pylori* infection are needed to better understand the immune responses against this bacteria.

Although several immunodominant epitopes of *H. pylori* protective antigens were identified (Chen et al., 2013; Yang et al., 2013; Li et al., 2015; Hu et al., 2016), much remains unclear concerning CD4^+^ T cell response to many *H. pylori* antigens. Our previous studies constructed an epitope vaccine based on our identified two Lpp20 H-2^d^ restricted CD4^+^ T cell epitopes (Li et al., 2012), which stimulated prophylactic and therapeutic responses with Th1-type profile against *H. pylori* in mice (Li et al., 2015). The MHC loci are genetically variable in mammals so that the highly polymorphic nature of MHC loci causes the immune systems of different subjects/species to focus on different epitopes/antigens within the same antigen/pathogen. This is the reason that many candidate vaccines succeed in murine experiments but fail in human clinical trials. Therefore, we were committed to mapping Lpp20 HLA-restricted epitopes. In the present study, to be able to efficiently identify immundominant Lpp20 epitopes, a short-term *in vitro* T cell expansion approach was used to increase the frequency of Lpp20-specific T cells in an unbiased fashion. This identifying analysis also required large arrays of overlapping synthetic peptides. However, the method was accurate and reliable. Moreover, this approach enabled us to evaluate whether these identified epitopes were immunodominant or subdominant.

In this study, the frequency of Lpp20-specific CD4^+^ T cells in *H. pylori*-infected subjects were observed higher than that in uninfected subjects, indicating that a systemic immune response can be elicited during *H. pylori* infection. Indeed, a very low-level Lpp20-specific CD4^+^ T-cell response was detected in some samples from non-*H. pylori*-infected patients. We believed that this was because of false-negative diagnosis of subjects who might have had brief or mild *H. pylori* infection. Then we used a systematic screening approach to evaluate the magnitude and extent of Lpp20-specific CD4^+^ T cell responses in *H. pylori-*infected individuals. The Lpp20-specific CD4^+^ T cell response mainly focused on two 18mer peptides (L_55–72_ and L_79–96_) on average, consisting with the widely observed immnuodominant phenomenon. Our previous studies identified two Lpp20 H-2^d^-restricted CD4^+^ T cell epitopes (L_58–72_ and L_83–97_) response to mice by SYFPEITHI prediction and 15mer overlapping synthetic peptides (Li et al., 2012). It is concluded that Lpp20-specific CD4^+^ T cell response in humans and mice seems to be almost the same. After that, the most potent core sequence of L_55–72_ and L_79–96_ were determined by 13mer overlapping peptides. They were L_57–69_ and L_83–95_, which stimulated CD4^+^ T cell response equivalent to the stimulation of L_55–72_ and L_79–96_, respectively.

The MHC loci are variable and highly polymorphic in mammals so that MHC alleles of the same/species generally focus on different peptides let alone MHC alleles from different species. For example, within the 11 reported CD4^+^ T cell epitopes from *H. pylori* UreB, including 4 mouse and 7 human epitopes, not a single one is recognized by both murine and human CD4^+^ T cell responses (Hu et al., 2016). Therefore, identification of a broad spectrum of immunodominant T cell epitopes across different HLA is required for the rational design of T cell epitope-based vaccine against *H. pylori*. However, only a few HLA-restricted CD4^+^ T cell epitopes of *H. pylori* UreB and HpaA antigens have been identified (Chen et al., 2013; Yang et al., 2013; Hu et al., 2016). The immunodominant epitopes were considerably different among individuals with different HLA alleles. The Allele Frequency Net Database (http://www.allelefrequencies.net/default.asp) shows that the frequency of HLA-DRB1^*^1501 in the Chinese Han population is relatively high (up to 10%) and the frequencies of HLA-DRB1^*^0803 and HLA-DRB1^*^1404 in the Chinese Han population are relative low (< 1%). However, HLA-DRB1^*^0803 is more common in some other populations, such as native populations in Papua New Guinea and Taiwan (up to 10%), Australia Aborigine (up to 20%). Chen et al. demonstrated that HpaA_88–100_-specific CD4^+^ T cell response was immunodominant in subjects expressing HLA-DRB1^*^1501 and HLA-DRB1^*^1501-restricted immunodominant CD4^+^ T-cell response to HpaA_88–100_ was related with reduced risk of severe *H. pylori*-associated gastric diseases (Chen et al., 2013). Interestingly, in the HLA-DRB1^*^1501 negative subject, HpaA_142–159_-specific CD4^+^ T cell response restricted by HLA-DRB1^*^0901 was the most immunodominant (Hu et al., 2016). In the present study, it was shown that L_57–69_ and L_83–95_ could induce dominant CD4^+^ T cell responses in subjects possessing HLA-DRB1 genotypes and the restriction molecules were detected by antibody blocking. Then, using a panel of B-LCLs with different HLA alleles as APCs to present peptides for CD4^+^ T cells, we found that L_57–69_ was restricted by HLA-DRB1^*^1501 and L_83–95_ was restricted by HLA-DRB1^*^1602. Moreover, the CD4^+^ T cells specific to these epitopes not only recognized autologous DCs pulsed with rLpp20 but also those loaded with HP-WCL, indicating that these epitopes are naturally processed and presented by APCs. Furthermore, L_57–69_ and L_83–95_ stimulated the proliferation of CD4^+^ T cells from *H. pylori*-infected subjects expressed HLA-DRB1^*^1501 and HLA-DRB1^*^1602, respectively. Therefore, HLA-DRB1^*^1501-restricted L_57–69_ and HLA-DRB1^*^1602-restricted L_83–95_ might be of important value for the development of novel vaccine against *H. pylori*. To further study the association of L_57–69_ and L_83–95_-specific CD4^+^ T cell responses with gastric diseases induced by *H. pylori*, HLA-DRB1^*^1501 and DRB1-1602^*^-expressing subjects with different gastric diseases, including atrophic gastritis, non-atrophic gastritis, antral gastritis pangastritis, peptic ulcer, duodenal ulcer, gastric cancer, will be assessed for L_57–69_ and L_83–95_-specific CD4^+^ T cell responses.

The knowledge gained in our study may not only help us to further understand the mechanism of protective immunity against *H. pylori* infection, but also help us to improve vaccine design. However, *H. pylori* has a large genome that encodes many protective antigens, such as UreaseB, UreaseA, Lpp20, CagA, VacA, and HapA. Thus, it is very important to better understand CD4^+^ T cell responses to other *H. pylori* encoded antigen at a population level and to identify other HLA-restricted immunodominant CD4^+^ T cell epitopes. In the future, we will construct subunit vaccine, which includes CD4^+^ T cell immunodominant epitopes of many *H. pylori* protective antigens, to induce Th1-type protective immunity.

## Ethics statement

The protocol was approved by the Institutional Human Ethics Review Board of Southern Medical Hospital, Southern Medical University, Guangzhou, China.

## Author contributions

YN contributed and designed the majority of the work. JY, DW, and JW separated cells and screened overlapping peptides. ZC expressed and purified rLpp20. YqL, BH, and ML operated ICS. JL cultured bacteria. ZC and LN collected samples. YL contributed and designed the majority of the work, as well as writing this manuscript.

### Conflict of interest statement

The authors declare that the research was conducted in the absence of any commercial or financial relationships that could be construed as a potential conflict of interest.
